# Supplementary material for: Identification of Six Novel Proteins Containing a ZP Module from Nemertean Species
Source: Biomolecules. 2024 Dec 2;14(12):1545. doi: 10.3390/biom14121545 (PMC11673550; doi:10.3390/biom14121545)
Supplement: Supplementary file 1 [file biomolecules-14-01545-s001.zip › biomolecules-3319061-supplementary.pdf]

```
                 SP
K.albo           MLLLSLLVVTCSLYAIEG---IAVGDACTNSTYCNYEGTDFVDHTKCDSSQCLCTTLYYG 57
K.fulv           MLLLSLLVATCSLYTIEAASGIAVGDACTNSTYCNYEGTASVDHTKCDASTCQCTSLYYG 60
                 ********.*****:**.   ******************  *******:*  * **:****

K.albo           ETGDTTCIQATVEGSCAGKTDAAGFCATADADAVCTSDACACNTGFEQYTSDVDGKEHCR 117
K.fulv           ETGDTTCIQATVGGSCAGKTDAAAFCATADADAVCTSDTCACNTGFEQYTSDVDGNEHCR 120
                 ************ **********.**************:***************:****

K.albo           KIEVGTSCDSSSGGDAFCQTRVAAFTCNVPTQTCECPSGETYFNDDCEPESFVAACNTCR 177
K.fulv           KIEVGTSCDSSSGGDAFCQTRVAAFTCNVPTQTCECPSGETYFNDDCEPESFVAACGTCR 180
                 ********************************************************.***
                                                                       EGFL
K.albo           ASGGHCYDVEDDGTPEGCSCPPSKQSSGADAAGTLTAGCDEGLANIGDYCDADGTPVGCV 237
K.fulv           ASGGHCYDVEDDGTPEGCSCPPSKQSSGSNAAGTLTAGCDEGLAHIGDYCDADGTPVGCV 240
                 ****************************::**************:***************

K.albo           GDHVTCVHAGGLDNRFTCECLPGYHAIPVGKEKKCYISIAKETDPNCQWCQQRGGVCYDT 297
K.fulv           GDHVTCVHAGGLDNRFTCECLPGYRAIPVGKEKKCYISIAKETDPNCKWCQQRGGVCYDI 300
                 ************************:**********************:***********
                                                   ZP
K.albo           DDDDIRDGCMCPASKSTSDEHDPQKSCDTDHVAVTCAASTMQVCYTPHNNAKYGDVAAKI 357
K.fulv           DDDDIRDGCMCPASKSTSDENDPSKSCDTDHVAVTCAASTMQICYTPHNNVKYGDVAAKL 360
                 ********************:**.*****************:*******.********:

K.albo           TAKEAYVYIQDHLGQDPCRFSAVSSDYCVTLNLATGETDACSTSVTYPSHRTINYNNWMV 417
K.fulv           TAKEAYVYIQGHLGQDPCRFSAVSSDYCVTLNLATGETDACSTSVTYPSHRTINYNNWMV 420
                 **********.*************************************************

K.albo           LQTSELGRSEKDATVHVYCQYDTLTHTFTSSGSQVIGFASIASEGEEVHPALDMDVEDEF 477
K.fulv           LQTSELGRSEKDATVHFYCQYDTLTHTFTSSGSKVIGFASVASEGQEVHPALDMDVEDEF 480
                 ****************.****************:******:****:**************

K.albo           GRDATIQGIRLGQPTRLIVEMTDDKSVYHSVRPQVCIASNRPELADPATSSILLLYDACP 537
K.fulv           GRDATIQGIRLGQPTRLIVEMTDDKSVYDSIRPEVCIASNRPELADPATSSILLLYDACP 540
                 ****************************.*:**:**************************

K.albo           VDNGYLNVRNMFNPDPADSEVSKTGLFPMFQFEDSDYVFFHCAVKVCRNEMDCSAPTCTL 597
K.fulv           VDNGYLNVRNTFNQDPADSEISKTGLFPMFQFEDSDYVFFHCAVKVCRNDMDCSAPTCSV 600
                 ********** ** ******:****************************:********::

K.albo           KKRSVGSPAETLERHVRELANDDVQLESLLSGPVKILRDNPMTGGKFVTDKKTIKDIFGI 657
K.fulv           KKRSVGSPAEILERHVRELANAEVPLESLLSGPVKLLRDDPMTGGKFVTDKKTIKDIFGI 660
                 ********** ********** :* **********:***:********************
                     TMD
K.albo           PRKYTIMIAIFAAVILVAIVLAVAVGVKRRRQSPPIRKRLDHGREKVTFKA         708
K.fulv           PRKYTIMIAIFAAVILVAIVLAVAVGVKRRRQSPPIRKRLDHGREKVTFKA         711
                 ***************************************************
```

Supplementary Figure 1. Alignment of NeZPL1 from *K. alborostrata* (K.albo) and *K. fulva* (K.fulv). EGFL; EGF-like domain, SP; signal peptide, TMD; transmembrane domain, ZP; ZP module. A CFCS is enclosed in a black box. The symbols below each residue shows similarity of both residues.

```
                SP                           CUB
K.albo          MHYFGLHGVVLCLLLRIPNVLARLETFEIDIQASVNNATISSPNYPSPYPTDVRYTWSIS 60
K.fulv          MHYFGLHGVFLCLILRIPNVLARLETFEIDIQASVNNATISSPNYPSPYPTDVRYTWTIS 60
                *********.***:*******************************************:**

K.albo          GPPGSRIAFYFDPVFEVEIEDETCYDYVKIHDGSSIRDVLIGEYCSFTKPESLDSTTYTN 120
K.fulv          GPPGSRIAFYFDPVFEVEIEDEICYDYVKIYDGSSARDVLIGEYCSFTKPESLDSTTYTN 120
                ********************** *******:**** ************************

                                                      CUB
K.albo          ELLVTFYSDDRVTFKGFEMTYWLIIDSTPLSEACDGSGRGVNLTMSDWPGTIFSPGYESH 180
K.fulv          ELLVTFYSDDRETFKGFEMTYWLIIDSTPLSEACDSSGQGVNITLSDWPGTIFSPGYESQ 180
                *********** ***********************.**:***:*:**************:

K.albo          GYDPLTHCIWRLERPVEGLIVIEFDPGRGKFDIGLTRDCTGDYIQVTCGSNTTDVHGPFC 240
K.fulv          GYDPLTHCIWRLERPVEGLIVIEFDPGRGKFDIGLTRDCTGDYVQVTCSSNVTDIHGPFC 240
                *******************************************:****.**.**:*****

                                                                  CUB
K.albo          GRNAPSVFIGRGDAAIVEFKTDAADGGEKGFRMQYFLVSGSAPSYGNMKFLQAELTVNSI 300
K.fulv          GRSAPSIFIGRGNAAIVEFKADAADGGEKGFRMQYFLVSASATSYGNMKFLQAETTVNSI 300
                **.***:*****:*******:******************.**.*********** *****

K.albo          LSHAGFGVTNYPLDTHCHWFIYAPIGMAIKYSFDPLFGIEDTHKCEFDYLEILDEEKMYS 360
K.fulv          QSHAGFGVTDYPLEVHCHWLIYAPIGMAIKYSFDPLFGIEDTHKCEYDYLAIFDEEKTDS 360
                 ********:***:.****:**************************:*** *:****  *

K.albo          YCGGTAPAGAVSNGDTLLITFVSDEFYSYQGFKIYYQISESTFETTLTTTLPPAPTPSSD 420
K.fulv          YCGGTAPAGAVSNGDTILITFMSDEFYSYQGFKIYYQISESAFETTPTPTLPPAPTPSSD 420
                ****************:****:*******************:**** *.***********

                       CUB
K.albo          PCSNPPMLVHAQENRTVYFSLPGYPNAVYNNDLSCEWFVLPSIDKSFSLYFAGDFSIEYT 480
K.fulv          PCSNPPMLVHAQENRTVYFSPAGYPNKVYDNDLSCEWFVLPSIDKPFSLYFAGDFSIEYT 480
                ******************** .**** **:***************.**************

K.albo          IGCVKDSVNVHAGINISAPIVGVHCGDQRPQSYFVSSAAAIHFHSDDNFIVGRGFQFALS 540
K.fulv          IGCVKDSVDIHSGINISAPIVGVHCGDQRPQSYFVSSAALIHFHSDDNFIVGRGFQFALS 540
                ********::*:*************************** ********************

                           CUB
K.albo          YDISDTACRYAPLVITMNEDPVTISTRNHPGNYPNGELCVWIFKSPPGTHMAYKIFDENY 600
K.fulv          YDISDTACRYAPLMITMNEDPVTISTRNHPGNYPNGELCVWIFKSPPGTQMAYNIFDENY 600
                *************:***********************************:***:******

K.albo          GIENSEDCLKDFLRVDDGLEESPNTIGQFCSTKPPFEVTSSDNYLRVTFSSDGAVYSEGF 660
K.fulv          GIENSEDCLKDFIRVDDGLEESPNTIGQFCGTKPPFELTSSDNYLRVTFSSDGAVRSEGF 660
                ************:*****************.******:***************** ****

                              CUB
K.albo          SMEVRLVDDKVSGACEGEEPLHVNTDTIITTPGYPNVYGENLICRWLVSSSTYAIDGAFE 720
K.fulv          SMEVRLVQDKISGACEGEEPLHVITDTIITTPGYPNAYGENMTCRWLVSSPTYAIDGAFE 720
                *******:**:************ ************.****: *******.*********

K.albo          PVYGLNDSSPCSGDRIDLIEWHNGTNGVPDPVNIGIHCGDQAPANFKTSGQKLLVEFRSD 780
K.fulv          PVYGLNDSSPCSGDRIDLIEWHNGTDGDPDPVNIGIHCGDQAPANFKTSGQKLLVEFRSD 780
                *************************:* ********************************

                                                CUB
K.albo          GSGPGLGFRLNLTAGHHPACGPDQPLEVEVGLDPVIVSPPIDTFSGEYFDNMECSWKMKT 840
K.fulv          GSGPGLGFRLNLTTGHHPACGPDQPLEVEVGLDPVIISPPIDTYSGEYFDNMECSWKMKT 840
                *************:**********************:******:****************

K.albo          KRAYRVMNASVLSTDMEDSASCSRDAAKLFDGPSTSDTLLSQSCGEGTPLLNFSSTGNEM 900
K.fulv          KRAYRVMNASVLSTDMEDSASCSRDSAKLFDGPSTSDTLLSKSCGAGTPLLNFSSTGNEM 900
                *************************:***************:*** **************
```

```
                                        CUB
K.albo    LVKFSSDESGGGGGFRMQFQEYLPPASAPCERMVGELWAEFYQQDFTSFNYPQEYSNYMN 960
K.fulv    LVKFSSDESGRGGGFRMQFQEHLPPASAPCERMIGELWAEFYEQHFTSFNYPQEYSNYMN 960
          ********** **********:***********:********:*.***************

K.albo    CTWIIKTRVGLLIQFNFSSVFDIETSSSCANDYVAIYDGYNDTAPVMIQHCGDSAPSNTY 1020
K.fulv    CTWIIKTRVGLLIQFNFSSVFDIETSSSCANDYVAIYDGDNDTAPVLIQHCGDSAPSNTY 1020
          *************************************** ******:*************

K.albo    VTTGKSARVVFVSDSENVATGFRLGYIALGQDSFSISEDLNNGTNGAKSDLNQPEDISAI 1080
K.fulv    VTTGKSARVVFVADSENVATGFRLGYIALGQDSFSFSDDLEPRTNESKSDLDQPEDISAI 1080
          ************:**********************:*:**:  ** :****:********

K.albo    SFTIFIWLNMESFYTTQFVLAEWTSPGQFALYVDTENQVVFHLRMSDNSDVIVRGGDLAS 1140
K.fulv    SFTIFIWLKMESFYTTQFVLAEWTSPGQFALYVDTENRVVFHLRMSDNSDVIVRGGDLAS 1140
          ********:****************************:**********************

K.albo    CDRWQFLAVTWDRVTSTAKVYVNGYVIGSATAAGGTKDLASSAAGTWQVGYNIQKDGEEF 1200
K.fulv    CDRWQFLAVTWDRVTSTAKVYLNGYVIGSATAAGGTKDLASSDTGTWQVGYNIQRDGETF 1200
          *********************:******************** :**********:*** *

K.albo    AGNLKDFKLFKSAFLTSEILRIAREDPTLFSDAILGGNCSVHVDCNLGFDPKKIHYSMCT 1260
K.fulv    AGSLKDFKLLKSAFLSSEILRIAREDPTLFSDAILGGNCSVHVDCNLGFDPKKIHYSMCT 1260
          **.******:*****:********************************************
                                      EGFL
K.albo    DQKICECVYGYHGNFGSCICRPIVLGTYCSHRNNPDDFCESGVKHSRCGSDGKCVCDPGY 1320
K.fulv    DQKICECVYGYHGNFGSCICRPIVLGTYCSHRNNPDDFCESGVKHSRCGSDGKCVCDPGY 1320
          ************************************************************

K.albo    YRQLNNWQQEYCEFYQPYSPCNDTVDCLTGMSRSSCIDGECFCPDGEVFIRSRKACESHS 1380
K.fulv    YRQLNNWQQEYCEFYQPYSPCNDTANCLTGMSRSSCIDGECFCPDGEVFIRSRKACESHS 1380
          ************************.:**********************************
                                                                 EGFL
K.albo    IVPDCKACRYSSGMCYDQDNDGTPDDCECPAYKVASDPDDWKIGCDIGIVEPEKFCDPKG 1440
K.fulv    IVPDCKACRYSSGMCYDQDNDGTPDDCECPAYKVASDPDDWKIGCDIGIVEPEKFCDPKG 1440
          ************************************************************

K.albo    KFSVCRSDHAECKPANVITGQYQCLCELGYTQVPAKPNPDYVYDCVKIINKHVDINCHWC 1500
K.fulv    KFSVCRSEHAECKPANVITGQYQCLCELGYTQVPAKPNPDYVYDCVKIINKHVDINCHWC 1500
          *******:****************************************************
                                                                    ZP
K.albo    DLRGGICVDRDLDDLRDECICPGTKSSTPFEIQHSKYDCDINYVDVQCGWPMMTVCYTPQ 1560
K.fulv    DLRGGICVDRDLDDLRDECICPGTKSSTPFEIQHSQYDCDIDYVDVQCGWPMMTVCYTPQ 1560
          ***********************************:*****:******************

K.albo    NHTKYFFLEDELEPYEYKVFVKYKQETPECWFKEVDGKHCLTLSLDSDMLGMCDVRKRIL 1620
K.fulv    NHTKYFFLEDELEPYEYKVFVKYKQETPECWFKQVDGKHCLTLSLDSDMLGMCDVRKRIL 1620
          *********************************:**************************

K.albo    PRQTAYYVSIRIQRTNFHWNYQDYTFYVYCKYDDVVDTWQGGIETGPIDVDSVGEMIKPK 1680
K.fulv    PRQTAYYVSIRIQRTNFHWNYQDYTFYVYCKYDDIGDTWRGGIETGPIDVDSVGEMIKPR 1680
          **********************************: ***:*******************:

K.albo    VVMNAYDKFGQTARVVKYGEQIQLIIELTDRQDIYSGIMVLMCIASSSGSLGDPASQTML 1740
K.fulv    VLMNAYDKFGQTARVVKYGDQIQLIIELTDRQDIYSSIKVLMCIASSSGSLGDPASQTML 1740
          *:*****************:****************.* *********************

K.albo    LIEDGCPVSDYITHFRQDPSNKRRASTSLFEIVSFEGSEALYYHCAVRVCHLNQNCHESA 1800
K.fulv    LIEDGCPVSDYITHFGQDPSNKRRASTSLFEIVSFEGSETLYYHCAVRVCRLNQNCQEST 1800
          *************** ***********************:**********:*****:**:
```

```
K.albo          CRSRRSLQSSRRPKEVYGYGEEEIFSSVSPLILVRRSVADVSVDRISDKDCVKFIGFDVC 1860
K.fulv          CRSRRSLKSSRRPEEVYGYGEEEIISSVSPLIRVRRSMADVSADGISDKDCVKFIGFDVC 1860
                *******:*****:**********:******* ****:****.* ***************

                        TMD
K.albo          NRSTRDILTVTLVSSCAIVLIALLLGVTIWYGRKSRTVEIEDSDDYNVDSIVLKYLQNLA 1920
K.fulv          NRSTRDIVTVTLVSSCAIVLIALLLGVTIWYGRKTGTVEIEESDDYNVDSIVLKYLQNLA 1920
                *******:**************************: *****:******************

K.albo          EDIPSDPDLDDLPPFEGMLANLNVPVIPEGGTLHNSDVDMDSLYAF               1966
K.fulv          EDIPSDPDLDDLPPFEGMLANLNVPVIPEAGTLHNSDVDMDSLYAF               1966
                *****************************.****************
```

Supplementary Figure 2. Alignment of NeZPL2 from *K. alborostrata* (K.albo) and *K. fulva* (K.fulv). CUB; CUB domain, EGFL: EGF-like domain, SP; signal peptide, TMD; transmembrane domain, ZP; ZP module. A CFCS is enclosed in a black box. The symbols below each residue shows similarity of both residues.

```
                    SP                                   VWD
K.albo      MELQRLIVLMVAIFIACFPISHVEGSCKNEAYADIVFLLDGSGSVSLDNFNKTKDFVKMM 60
K.fulv      MELQKLIALMVAIILACIPIPHVEGSCKNEAYADIVILLDGSGSVTLDNFNKMKDFVKIM 60
            ****:**.*****::**:**.***************:********:****** *****:*

K.albo      IDNIEVGPLGSLVGVVVFSSSVTFSQSFVPTSTAEDIKTLVDGLTYPSGGTATSAGLEGV 120
K.fulv      IDNIEVGRLGSLIGVIVYASSVTFSQSFVPTSTAEDIKTLVDGLIYPGGGTGTSAGLEGV 120
            ******* ****:**:*::************************* **.***.********

K.albo      RIMFLDSNARSDAPDIAYVLTDGQSNDPTATASKAQEIRDAGAKIYCLGIGGADRGEIIA 180
K.fulv      RTMFLDSNARSDAPDIAYLLTDGISNDPAATASKAQEIRDAGAKIYCLGIGGADLSEIIT 180
            * ****************:**** ****:*************************  .***:

K.albo      IASSPAQNYSAFIESFEDLQDVLDDLVSATCNIPQLVKWCEVHIDCNDGTDTNSLAYTKC 240
K.fulv      IASSPAQNYSAFLETFEELPDVLDDLVSATCNIPQLVKWCEVHIDCNDGADTNSLAYTKC 240
            ************:*:**:* *****************************:**********

K.albo      ENKKCVCVDGYYQDTRQESCKPITIGTSCEEETGPDAFCEAGLAHSHCDVDGSTGCKCDD 300
K.fulv      ENKKCVCVDGYYQDTRQESCKPITIGTACEEDTDPNAFCDAGLAHSHCDVDGSTGCKCND 300
            ***************************:***:*.*:***:******************:*

K.albo      GYHTELYNWGQVYCKHYQPGSDCSSDSDCEPGMTSGICVNNECECPSGQVYVQRSEACET 360
K.fulv      GYHTELNNWGQVYCKHYQPGSDCSTDSDCETGMTRGVCVNSECECPSGQVYVQGSEACET 360
            ****** *****************:*****.*** *:***.************ ******

K.albo      PLFTTDCDPCTSSGGVCYDSATGTSPDVCGCPDYKRSSDPDDFTVGCDVSLVDPEIMCDP 420
K.fulv      PLFTTACDPCTSSGGVCYDSATGTSPDVCGCPDYKRSSDRDDFTVGCDVSLVDPEILCDP 420
            ***** ********************************* ****************:***

K.albo      DGVLSVCRSANAECLHAKHISGHSQCQCKLTYAQVPERFNPDYNAKCVKAISLFTDPNCQ 480
K.fulv      DGVLSVCRSANAECLHAKHISGHSQCQCKLTYAQVPERFNPDYNAKCVKAISLFTDPNCQ 480
            ************************************************************
                                                                 ZP
K.albo      WCDRRGGICVDLDGDFLRNDCMCPATMSSSSHAHPKYDCDTEHANVTCTKDSMTVQYFPH 540
K.fulv      WCDRRGGICVDLDGDFLRNDCMCPATMSTSSHAHPKYDCDTEHANVTCTKDSMTVQYFPH 540
            ****************************:*******************************

K.albo      NNTKFQTLKSDLLEEDGKVFVMGKQKSGECWFTKTGDHYELTLPLHDEKFAMCGHRKIES 600
K.fulv      NNTKFQTLTSDLLEEDGKVFVMGKQKSGECWFTKTGDHYELTLPLHDEKLAMCGLRKIES 600
            ********.****************************************:**** *****

K.albo      PDASTSYKTEVRVQRGRTGERIKSWSDFTFGVYCTSVDLYEVGRGGVLVTDPDISTSSAS 660
K.fulv      PDASTPYKTEVRVQRGRTGERIKSWSDFTFGVYCTYVELNEVGHGGVLVTDPDITTSSAS 660
            *****.***************************** *:* ***:**********:*****

K.albo      EGKRVQAELSIEVVDKFGQDAAQSGIGQGDNVQLKVIMSEDAEAYVSIKVETCLAADRPN 720
K.fulv      EGKRVQAELSIEVVDQFGQDAAQSGIGQGDNVQLKVLMSEDAGAYISPKVETCLAADRPN 720
            ***************:********************:***** **:* ************

K.albo      LDDPRVQTQLLVDNGCPVGLSEPSFELDPTDPTTALSEYFPTVSFDGSAFIYFHCAIRVC 780
K.fulv      RDDPRVNTQLLVDNGCPVGISEPSFELDPTDPKMALSEYFPTVSFDGSAFIYFHCAVRVC 780
             *****:************:************. **********************:***

K.albo      RQSSQCDATVCTRKRRSLESNVRSQGNLLDNAEFNWGVRSGASSKAIPVRGRDDDADVTY 840
K.fulv      RQSSHCETSVCNRKRRSLESNVRSQGDLLDNAEFDWGVRSGASSKAIPVRGRDDDVDVTY 840
            ****:*:::**.**************:*******:********************.****
```

```
                                   TMD
K.albo         KTDKECPGFLGVCGFNETTFASMLAGIGVVLTIAVTIAILVRRGRLMRMKEEFSTAAQVE 900
K.fulv         KTDKECPGFLGACGFSETSFATMLAGIGVVLTIAVTIAVLVRRGRLMRMKEEFSTAAQVE 900
               ***********.***.**:**:****************:*********************

K.albo         KMKMSWPYSSQTPCHIPPGEFIRRV                                    925
K.fulv         KMKMSWPYPSQTPCHIPPGEFIRRV                                    925
               ********.****************
```

Supplementary Figure 3. Alignment of NeZPL3 from *K. alborostrata* (K.albo) and *K. fulva* (K.fulv). SP; signal peptide, TMD; transmembrane domain, VWD; Von Willebrand factor type D domain, ZP; ZP module. A CFCS is enclosed in a black box. The symbols below each residue shows similarity of both residues.

```
           SP
K.albo     MRLRLLFTTMTLLSWTQAILVGDACTNHTFCNYEGSAPLDHTYCLSGTCQCTSLYFGATG 60
K.fulv     MMLRLLFTTMTLLSWTQAILVGDACTNHTFCNYEGSAPLDHTYCLSGTCQCTSLFFGATG 60
           * ****************************************************:*****

                                                                   EGFL
K.albo     DSICRMANIGDACTDEAFCDTACGNAECSSGVCVCSLGTYESMGTFGKEVCRKTTIGTSC 120
K.fulv     DSLCRMANIGDACTEEAFCDTACGNAECSSGVCACSLGTYESISTFGKEVCRKTTIGTSC 120
           **:***********:******************.********:.****************

K.albo     EDDAFCSLGITNSICDPLSKTCVCASGETYVDGDQLCRGESYVSPTCDVCRSTGGHCFDV 180
K.fulv     EDDAFCSLGITNSICDPLSKTCECASGETYVDADQLCRGESYVSPTCDVCRSTGGHCFDV 180
           ********************** **********.**************************

                                                    EGFL
K.albo     DGDGTPEGCACTTSKKSSGTNEAESLTYGCDLGLAEIGDYCDKDGTPIVCKQPNSICSHA 240
K.fulv     DGDGTPEGCACTTSKKSSGTSEAESLTYGCDLGLAEIGDYCDKDGTPIVCKQPNSICSHA 240
           ********************.***************************************

K.albo     GELSNQYSCECGPGYFPVVSKYSKDTYLCYHALSEYTDPNCQWCNERGGTCYDIDEDDIR 300
K.fulv     AELSNSYSCECGPGYFPVVSKYSQDTYLCYHALSEYTDPNCQWCNERGGTCYDIDEDDIR 300
           .****.*****************:************************************

                                                  ZP
K.albo     EGCICPATRSTNEEYIHNPREDCNVLHFTLQCQALQMRMCYTPHNTTLLPNMTHLLDAQE 360
K.fulv     EGCICPATRSTNEEDIHNPREDCNELHLTLQCQTLQMRMCYTPHNTTLLPNMTHLLDAQE 360
           ************** ********* **:*****:**************************

K.albo     AYIYIRDHHGKDPCIFEKQDDGDWCLDLSLGNNYTDACGTVVTYPSENEISYQTWLVLQM 420
K.fulv     AYIYVRDHHGKDPCIFEKEEGGDWCLDLELGQNDTDACGTVVTYPSENEISYQTWLVLQM 420
           ****:*************::.********.**:* *************************

K.albo     SGSQRTNDDLFFHAYCDYDTLVHTVTASGTEVVGFASISSEGHKIKPDLVMNVKDEFGRD 480
K.fulv     SGSQRTNDDLFFHAYCDYDTLVHTVTASGTEVVGFASISSEGHKIKPDLVMNVNDEFGRD 480
           *****************************************************:******

K.albo     GAQQGIRLGHPASIEITMLDNKNVYTGVRPEVCIASDRPELGHPNAKTVVLIYNGCSMGS 540
K.fulv     GALQGIRLGQPASIEITMLDNKNVYTGVRPEVCIASDRPELGHPNAKTVLLIYNGCSMGT 540
           ** ******:***************************************:*********:

K.albo     EIMKMTFFVDPTDSGKWTTRPFQMFQFEGSEFMFFHCAVKVCRITSECAEKTDCSIERRK 600
K.fulv     GTMKMAFFVDPTDSGKWLTRPFRMFQFEGSEFMFFHCAMKVCRIASECAEKTDCSNERRK 600
             ***:*********** ****:***************:*****:********** ****

K.albo     RASGIE-SQGLVSRVARSPGQSSRNDDPLQSLQSAPIRILDEDSYLPGSNGGRYERTNTK 659
K.fulv     RASGLGDSQGPVSRAARSPGRSSKNDDPLQSLQSAPIRILDEDSYLPGSDGGPLERSKTK 660
           ****:  *** ***.*****:**:*************************:**  **::**

                     TMD
K.albo     LSGLFSRHGPLLIGIIGGVLSLFIILVATLTFKLKIRPDITKNKRETKQPLVL       712
K.fulv     FSGLFNRHGPLLIGIIGGVLSLFIILVAALTYRFKIRPDMTKNRRETKQPLVL       713
           :****.**********************:**:::*****:***:*********
```

Supplementary Figure 4. Alignment of NeZPL4 from K. alborostrata (K.albo) and K. fulva (K.fulv). EGFL; EGF-like domain, SP; signal peptide, TMD; transmembrane domain, ZP; ZP module. A CFCS is enclosed in a black box. The symbols below each residue shows similarity of both residues.

```
                    SP                                            EGFL
K.albo      MVYVKGKSSPGVFAAMESLWVLFFFLSPRVLTLDVKCSTVGCPCTLHADCNDVHLGEQSL 60
K.fulv      MVYVIGKSTPGVFATMESLWIFVFFLSPRVLTLDVKCSAVGCPCTLHADCNDVHLGEQNL 60
            **** ***:*****:*****::.***************:*******************.*

K.albo      LNSLCIDRKCACVEGYIGSVGSTICERVRLGAVCSPNDVLDDDDYCNKNVPNSVCDKGAG 120
K.fulv      LNSLCIDNKCACVEGYIGSVGSTICERVRLGTACSPNDVLDADDYCNKNVPNSVCDEEAG 120
            *******.***********************:.******** **************: **

K.albo      ICTCGRDAYRSITTRGQEYCLCGYNQVYIQSLGACVDESFSTECEACKNTGGICYNIGQG 180
K.fulv      ICTCGRDAFRRATTRGQEYCLCGYNQVYIQSLRACVDESFSPECEACKNTGGICYHIGQA 180
            ********:*  ******************** ********.*************:***.
                                EGFL
K.albo      GVLG-CECPHERQTVVDVTSGCRRERVVLGERCDDDASCVTANAICDRTCQCNDGYAQYS 240
K.fulv      GVLDGCECPHERRTVVDVISGCRHERVVLGERCDNEASCVTANAICDRTCQCDDGYAQYS 240
            ***. *******:***** ****:**********::****************:*******

K.albo      SDNIVEFPVLCAKVINSSVDPNCHWCDSNNGSCIDMNGDYLRDDCVCPQTRRSSGSSHPS 300
K.fulv      SDNIVEFPVLCAKVINSSVDPNCHWCDSNNGSCIDINGDYLRDDCVCPQTRRSSGSSHPS 300
            ***********************************:************************
                                          ZP
K.albo      QDCDETLFEVSCSPDTMTTCYKPPENRRRSRDRRDRAEYKIVVKTRQKLPECVFRRKKLG 360
K.fulv      QDCDETLFEVSCSPDTMTTCYKPPEYRRRSRDRRDRAEYKIVVKTRQKLPECVFHRKKLG 360
            ************************* ****************************:*****

K.albo      VYCSRFQLMLAMLDVCDIHRDILNDMEVYSTVVAVHTPDFGNLRSPDSFSFTSFCKFDVK 420
K.fulv      IYCSRFQLMPAMLDVCDIHRDILNDMEVYSTVVAVHTPDFGNLRSPDSFSFTSFCKFDVK 420
            :******** **************************************************

K.albo      QKTASGGTDVISYTSKHGYNGEFNPRLRLVLKDERSKAIMDVFRPDRQLQLAVKLLDYKD 480
K.fulv      QKTASGGTDVISYTSKHGYSGEFNPRLRLALKDERSKAITDVFRPDRQLQLAVKLLEYKD 480
            *******************.*********.********* ****************:***

K.albo      IYSSIKVDLCVVSTSSNTSDIDAIISTLISGGCPSSGIPNFVHHQQDRRTMETALFFLPT 540
K.fulv      IYSSIKVDFCVVSTSSNTSNLDAIISTLISGGCPSPGMANFVHHQQDRRTMKTALFFLPT 540
            ********:**********::**************.*:.************:********

K.albo      SETFERVFIHCAVRVCRQDGGCSPSDCDRRRRSESNGALNEMMTGKSRDWGVHSGVTSQG 600
K.fulv      SETFGRVFIHCAVRVCRQDGGCSPSDCDRRRRSESNGALNEMMTGKSRDWGVHSGVTSQG 600
            **** *******************************************************

K.albo      ILILPPLVEVTTPIVSLWLKDKTKNHISKKVIPNKDEKKKLDHAGRPETKDEFEEQQTPP 660
K.fulv      ILILPPLVEVTTPVVSLWLKDKAKEPIRKKVIPNSDGEKKLDNAGLPETKDEFEEQQTPP 660
            *************:********:*: * ******.* :****:** **************
                                TMD
K.albo      DHIKDCDGLFGSCKFKDLHLILTAATLVIIFIVVASLTYFIYHRRIRSLEDEINHLSGTP 720
K.fulv      DHIKDCDGLFGSCKFKDLHLILTASTLVIIFIVVASLTYFIYHRRIRNLEDEINHLSGTP 720
            ************************:**********************.************

K.albo      VKSGTTVTWTTPGAYRQYWSDSEPSSYI                                 748
K.fulv      VKSGTTVTWTTPGAYRPYWSDSEPSSYI                                 748
            **************** ***********
```

Supplementary Figure 5. Alignment of NeZPL5 from K. alborostrata (K.albo) and K. fulva (K.fulv). EGF-L; EGF-like domain, SP; signal peptide, TMD; transmembrane domain, ZP; ZP module. A CFCS is enclosed

in a black box. The symbols below each residue shows similarity of both residues.

```
                     SP
K.albo               MLGGIIVVLAVAFGSCNVKAILVGTPCTSHKFCNFDGPEAIPQTKCDPLTVTCQCTILYY 60
K.fulv               MLVGIIVVVAVAFTSCNVNAILVGTPCASHKFCNFDGPEAIPQTKCDPSTATCQCTILYY 60
                     ** *****:**** ****:********:******************** *.*********
                                    EGFL
K.albo               GDANDTQCIQAQLEHACSAKEDPDRFCQTAAAHAICDDGSGTCQCENGYYAATVYGYRIC 120
K.fulv               GDANDTKCVQAQLGDSCSTKEDPDRFCQTAAPHAICDNGSATCQCENEYYAATVYGYRVC 120
                     ******:*:**** .:**:************.*****:**.****** **********:*

K.albo               KAVEIGSPCNASDPSSANICGLAVSNSECNSVKGSCQCPTDMIFVAATKECLRQISSPNC 180
K.fulv               KAVEIGSPCNASDPSSANICGLAVINSECNSVIGSCQCPTDMIFVAATKECLRHSSSPNC 180
                     ************************ ******* ********************: *****
                                                                        EGFL
K.albo               STCYRTSGHCFDREDDGTPSGCTCPLNRASTGSTDEDTLRIGCDFAYAQLGERCL---PG 237
K.fulv               STCYRTSGHCFDREDDGTPSGCTCPLNRASTGSTDEDTLRIGCDFAYAQLGERCLPDMPD 240
                     *******************************************************   *.

K.albo               GDSRILCNSKHAACIHAGYLNNKYKCSCRPGYYPVPSCKDDSVPAVCRLSISTTTDPNCR 297
K.fulv               GDNRIPCNAKHAECRHAGYLNNKYKCSCRPGYYPVPSCKDDSVPAVCRLSISATTDPNCR 300
                     **.** **:*** * *************************************:*******
                                                                                ZP
K.albo               WCISRGGVCYDVDEDGIRDGCHCPNSRSTSDETDPRNDCDVDHVSVECETTTMSVCYMPH 357
K.fulv               WCTNRGGVCYDVDEDGIRDGCHCPNSRSTSDETDPRNDCDVDHVSVECETTTMSLCYMPH 360
                     ** .**************************************************:*****

K.albo               NTRMLPDVATKLETGEVLLYVENYHGINPCMFKMTNSSGRSDWCLELDLRVSRMGFCGTE 417
K.fulv               NTRMLPDVATKLETGEVLLYVENYHGINPCMFKMTNSSGRSDWCLELDLRVSGMGFCGTE 420
                     **************************************************** *******

K.albo               MNYPKSNVISYSNTMVIQTSAEARTNNDLAVNLFCQYDTLVHAFTAMGTEVQLQQVAASE 477
K.fulv               MGYPTSNVISYSNTMVIQTSAAARTNNDLAVNVFCQYDTLVHAFTAMGTEVQLQQVAASE 480
                     *.**.**************** **********:***************************

K.albo               GVSVHPLLTINVYDQFGRDATFLGARIGDPIYLEVNMVDDKAVYDSFRPEVCIASSRPEL 537
K.fulv               GVSVHPLLTINIYDQFDRDATFLGARIGDPIYLEVTMVDDKAVYDSFRPEVCIASSRPEL 540
                     ***********:****.******************.************************

K.albo               SHPNAATIFLVYDGCPVHNSYLSVADNFMAFPNDGKMMRTGLFSMFKFEESNFVFFHCAV 597
K.fulv               SHPNADTIFLVYDGCPVHNSYLSVADNFMAGPNGGKMMRTGLFSMFKFEESNFVFFHCAV 600
                     ***** ************************ **.**************************

K.albo               KVCRRAAECTPFNCNSSRKRREIIDNRGRTPPESRDARDQPRDDDVIASLQSRAVPILGA 657
K.fulv               KVCRRAAECTPFNCNSSRKRREIIDNHGRTSQESRDARDQPRDDDVLAPLQSRAVPILGA 660
                     **************************:***. **************:*.***********
                                            TMD
K.albo               DETEEKDPGKPTRPGGGVFSPWLMVIVAIGGGIVSLMFLLLVVLACKVWLHRSSKDLPHQ 717
K.fulv               DETEENDPGKPIRPGGGLFSPRLMVIIAICGGIVSLMFLLLVVLACKVRLHRSRKDLPHQ 720
                     *****:***** *****:*** ****:** ****************** **** ******

K.albo               TSLRHDAFFKHEETQA-----                                        733
K.fulv               TSLRPIAKDTFFKQAIEETQA                                        741
                     ****  *  .. :
```

Supplementary Figure 6. Alignment of NeZPL6 from K. alborostrata (K.albo) and K. fulva (K.fulv). EGF-L; EGF-like domain, SP; signal peptide, TMD; transmembrane domain, ZP; ZP module. A CFCS is enclosed in a black box. The symbols below each residue shows similarity of both residues.

Supplementary Table 1. Primers used in this study.

primer name	sequence (5' to 3')
**for ISH probes**	
NeZPL1_F	GATTACGCCAAGCTTAGGCGACGCGTGTACGAACAG
NeZPL1_R	GACACGGACCATGTTGCTGTA
NeZPL2_F	GATACATTTCCATTCAGATGAC
NeZPL2_R	CTGAAACTGCATCCGGAAGC
NeZPL3_F	CACTAGGTCATCAAGAACGTC
NeZPL4_F	CCGCGTGTGAGCAGATTGAATTTGGC
NeZPL5_F	GGCACACACAATCATCTCGTAGG
NeZPL6_F	GATGTATACGTAAGCCTCTTTTGC
UPM (common reverse primer for NeZPL3-6)	CTAATACGACTCACTATAGGGCAAGCAGTGGTATCAACGCAGAGT
**for constructing EGFP-NeZPL6 fusion**	
Ka_ZP6F	ATCCTTGTTGGAACACCATG
Kf_ZP6F	ATCCTTGTTGGTACTCCATG
KaKf_ZP6R	ACATTGCATGAACCGAAAGC
KaGFP_ZP6F	CGGTTCATGCAATGTAAATGCAGTGAGCAAGGGCGAGGAGCTG
KfGFP_ZP6F	CGGTTCATGCAATGTAAATGCAGTGAGCAAGGGCGAGGAGCTG
KaGFP_ZP6R	TGTTCCAACAAGGATCTTGTACAGCTCGTCCATGC
KfGFP_ZP6R	AGTACCAACAAGGATCTTGTACAGCTCGTCCATGC
ZP6R	CTAATACGACTCACTGACACTAAGCTTGCGTCTCTTCG
